# Aberrant Complement System Activation in Neurological Disorders

**DOI:** 10.3390/ijms22094675

**Published:** 2021-04-28

**Authors:** Karolina Ziabska, Malgorzata Ziemka-Nalecz, Paulina Pawelec, Joanna Sypecka, Teresa Zalewska

**Affiliations:** Mossakowski Medical Research Centre, NeuroRepair Department, Polish Academy of Sciences, 5 Pawinskiego Street, 02-106 Warsaw, Poland; kziabska@imdik.pan.pl (K.Z.); mnalecz@imdik.pan.pl (M.Z.-N.); ppawelec@imdik.pan.pl (P.P.); jsypecka@imdik.pan.pl (J.S.)

**Keywords:** complement system, cerebral ischaemia, traumatic brain injury, spinal cord injury, neurodegenerative diseases, Parkinson’s disease, amyotrophic lateral sclerosis, Huntington disease, multiple sclerosis, epilepsy, schizophrenia, autism

## Abstract

The complement system is an assembly of proteins that collectively participate in the functions of the healthy and diseased brain. The complement system plays an important role in the maintenance of uninjured (healthy) brain homeostasis, contributing to the clearance of invading pathogens and apoptotic cells, and limiting the inflammatory immune response. However, overactivation or underregulation of the entire complement cascade within the brain may lead to neuronal damage and disturbances in brain function. During the last decade, there has been a growing interest in the role that this cascading pathway plays in the neuropathology of a diverse array of brain disorders (e.g., acute neurotraumatic insult, chronic neurodegenerative diseases, and psychiatric disturbances) in which interruption of neuronal homeostasis triggers complement activation. Dysfunction of the complement promotes a disease-specific response that may have either beneficial or detrimental effects. Despite recent advances, the explicit link between complement component regulation and brain disorders remains unclear. Therefore, a comprehensible understanding of such relationships at different stages of diseases could provide new insight into potential therapeutic targets to ameliorate or slow progression of currently intractable disorders in the nervous system. Hence, the aim of this review is to provide a summary of the literature on the emerging role of the complement system in certain brain disorders.

## 1. Introduction

**Figure 1:**
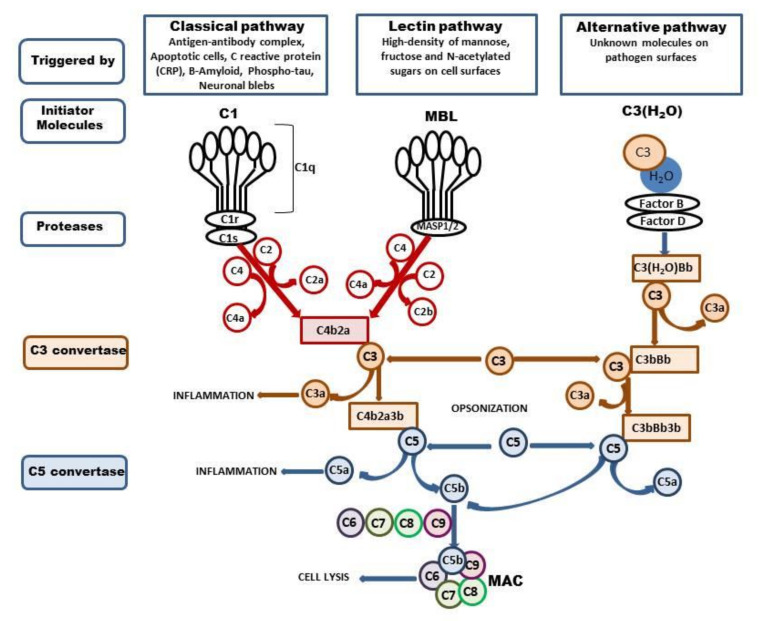
Activation of complement system. The complement system can be activated via three ways: classical, lectin, and alternative. The classical pathway is triggered by binding of the antibody-antigen complexes, apoptotic cells, CRP, Β-Amyloid, phospho-tau, or neuronal blebs to C1. The C1 is composed with C1q, C1r, and C1s, and binding of Cq1 with enumerated components changes the C1 conformation and activates C1s protease. The active C1s cleaves the C4 to C4a and C4b and C2 to C2a and C2, and forms C3 convertase—C4b2a which splits C3 into two fragments, C3b, which can opsonize microbial pathogens, and C3a, which activates mast cells and macrophages and promotes inflammation. The lectin pathway is activated by the binding of mannose-binding lectin (MBL) to mannose residues on the pathogen surface. This in turn activates the MBL-associated serine proteases, MASP-1/2, which activate C4 and C2 and form C4b2a. The C3b can bind to C4b2b to form the C5 convertase (C4b2a3b). C5 is cleaved to form C5a, which promotes inflammation, and C5b, which binds to C6, C7, C8, and C9 and forms the membrane attack complex (MAC) which enables cell lysis. The alternative pathway is triggered by hydrolysis of C3 to C3-H_2_O; then, factors B and D generate the C3 convertase—C3(H_2_O)Bb, which cleaves other C3 molecules to C3a and C3b. This in turn forms C3bBb complex which continues cleavage of the C3 and enables formation of the C5 convertase—C3bBb3b, which cleaves C5 and leads to the formation of the MAC.

The complement system is composed of a large family of circulating and membrane-associated proteins that act synergistically in a sequential cascade-like manner to execute and regulate its function. There are three different complement pathways initiated by different stimuli: the classical, alternative, and lectin pathways. All of them converge at component C3, a central molecule in the complement system that ultimately drives complement functions. The cascade involves more than 40 proteins, and the sequence of events was reviewed in great detail elsewhere [1,2,3,4]. Thus, we omit detailed general characteristics and confine ourselves to a brief summary of the current data (Figure 1). Most complement proteins are produced in the liver; however, extrahepatic production of the complement by several organs was also identified. In the brain, complement components are synthesized particularly by resident neurons and glial cells [5,6,7] or are carried by an influx of blood following injury-induced interruption of the BBB [8]. The precise role of the complement in the healthy brain is rather complex. One of the primary actions of complement activation within the central nervous system is a defense of neurons from potentially harmful toxic stimuli, such as aberrant protein aggregates and cell debris, through opsonization by component 3b. As an effector of innate immunity, the complement promotes inflammation via anaphylatoxin (C3a and C5a) and in the final stage initiates assembly to the membrane attack complex (C5b-9, MAC) [9]. In cooperation with other immune and physiological systems, the complement contributes to normal tissue development and to the proliferation, differentiation, and migration of neural progenitors [10,11]. Furthermore, the complement system plays an unexpected role in the elimination (pruning) of inappropriate synapses and mobilization of phagocytes to restore and maintain homeostasis [12,13].

Underregulated or overactivated systems may lead to unwarranted neuronal damage and disturbances in brain function. However, the molecular events that lead from the alteration of this initial complement cleavage to brain insult are not completely understood. Based on recent studies, alterations in complement-mediated synaptic remodeling are hypothesized to contribute to synapse dysfunction and decline in cognitive functions in several neurodegenerative and psychiatric disorders. As another option, complement activation fragments can induce local stimulation of microglia/astrocytes that together with other proinflammatory cascades accelerate pathogenesis and neuronal damage. However, despite recent advances, several aspects of the participation of the complement system in the pathogenesis of brain disorders remain unclear. The understanding of component functions at different stages of diseases could provide new insight into potential therapeutic targets.

Hence, the aim of this review is to provide a brief summary of the current knowledge about the functional role of complement regulation in selected neuropathologies, including ischemia, traumatic brain injury, spinal cord injury, neurodevelopmental impairments (schizophrenia and autism spectrum disorder), neurodegenerative diseases, and chronic disorders such as Parkinson disease (PD), Huntington disease (HD), amyotrophic lateral sclerosis (ALS), multiple sclerosis (MS), and epilepsy.

## 2. Complement in Acute Brain Trauma

### 2.1. Brain Ischemia

Brain ischemia triggers a widespread inflammatory reaction that, in conjunction with other induced responses, such as excitotoxicity and oxidative stress, contributes to neuronal death and neurological deficits. Inflammation, the key response to brain injury, is driven primarily by activation of inflammatory glial cells residing in the CNS together with infiltrating cells of the peripheral immune system to produce several proinflammatory factors, which lead to disruption of cellular homeostasis and structural damage to brain tissue. Based on several studies, it was postulated that the complement system represents one of the major effectors of the innate immune system, and it was assumed to play a prominent role as one of the putative mechanisms in the pathophysiology of ischemic stroke.

The well-established neurobiological role of the complement was defined in large part by clinical data. Clinical studies documented elevated levels of certain components, specifically C3, its conversion product C3a, and C5b-9, in plasma/serum samples taken from ischemic patients. Moreover, the presence of these factors likely predicts the risk of outcome [14,15,16,17,18]. It is worth noting that the presence of C1q, C4d, C3c, C9, and MAC/C5 was detected in the ischemic regions of stroke patients postmortem [15,19,20,21,22]. These findings, in conjunction with the reduced levels of the system regulators CD55 and CD59, indicate impairment of the whole system.

A functional role of the complement in experimental ischemia/reperfusion was demonstrated early by Huang et al. [23], who found that complement inhibitory depletion induced by sLex-glycosylated protein results in neuronal protection. Similarly, a protective effect was observed after cobra venous factor (CVF) pretreatment before the induction of transient ischemia in rodents. These animals present better outcomes in terms of somatosensory-evoked potential and reduced infarct volume [24]. Beneficial effects were also observed in neonatal hypoxia [25,26]. In contrast, another set of data revealed that CVF did not effectively reduce infarct volume in permanent ischemia or thromboembolic stroke [27,28].

The impact of the complement on the pathophysiology of ischemia was confirmed using transgenic mice deficient in specific complement proteins C1q, C3, and C5. Mice devoid of the C1q gene did not show any protection after ischemia induced in adult animals [20,29], despite the strongly upregulated expression of mRNA-encoding C1q in the brain following focal and global ischemia [30,31]. Simply stated, the lack of beneficial effects was not coupled with the activity of the classic pathway. The elevation of this component on neuronal cell bodies may imply its involvement in the effective clearance of damaged neurons or cellular debris [23,32,33].

Soon after the discovery of the lack of neuroprotection in C1q−/− mice, it became clear that other components respond to complement action in ischemia/reperfusion. Subsequent works performed on this matter focused on activation of C3, the central player in activation pathways. The level of component C3 is elevated after stroke and might participate in reperfusion injury [12,34,35]. Deletion of the C3 gene resulted in smaller stroke volume, diminished neutrophil infiltration, suppression of oxidative stress, and improvement of locomotor score outcomes after transient ischemia in mice. Furthermore, reconstitution with C3 protein before ischemia eliminated the effect [20]. These findings correspond with those of an in vitro study that showed that inhibition of C3 expression via small interfering RNA enhanced the viability of cultured neurons under OGD conditions [20,36].

The neuroprotective mechanism is dependent on C3a, the cleavage product of C3, which mediates downstream responses by interacting with its cognitive receptor C3aR. The generation of C3a is known to have proinflammatory properties, and its expression appears to be detrimental in several models of CNS injury. However, in some cases, e.g., following LPS administration, C3a presents anti-inflammatory action exhibited by decreasing LPS-mediated cytokine release [37].

Component C3a binds to its canonical receptor C3aR. Activation of C3aR in a mouse model of transient and permanent ischemia led to neutrophil recruitment to the ischemic zone and worsened the ischemia-induced tissue injury [38,39]. Furthermore, deletion or pharmacological inhibition of C3aR was found to improve functional and morphological outcomes following transient but not permanent ischemia in adult mouse models. This effect may be connected with restoration of post-ischemic blood flow to the brain [39]. Additional data showed that absence of the CR3 receptor protects against intracerebral hemorrhage induced by tPA treatment after MCAO. It is quite possible that reduced BBB permeability does not allow for massive neutrophil infiltration and suppresses infarction [40,41].

However, although an antagonist of CR3 presents a potentially protective action following ischemic injury, it has become clear that C3 and C3aR deficiency impairs ischemia-induced neurogenesis and consequently suppresses tissue repair following the insult [12,42,43,44]. Surprisingly, controversial data indicated improvements in functional recovery after intranasal administration of C3 in the post-acute phase of photothrombotic stroke [45]. It can thus be concluded that both C3 and C3aR have dual roles, and therapeutic manipulation of C3 must be carefully timed. An improved understanding of the time course of complement involvement may identify a therapeutic window during which complement deficiency will improve outcomes. Temporal distinction between the processes of brain injury and repair allows for the rational design of strategies.

Surprisingly, the role of the second convertase cleavage product, C3b, in ischemic stroke also remains largely undetermined. Research implies that C3b is involved in the elimination of pathogens or injured tissue [46], which may contribute to tissue repair and recovery after injury. Additionally, the function of one particular C3b receptor, CR1, is not well established in the human CNS [47,48,49,50,51]. Similarly, the role of CR2 in the brain was scarcely investigated until now.

The other intensively investigated component is C5, which is involved in a final step of complement system activation. C5 exerts activities after being cleaved by convertase 5 to soluble factor C5a and membrane-bound C5b [12,52,53]. C5a signals through the intracellular receptor C5aR1 (known as CD88). In general, the functional role of C5 in ischemic injury is unclear and strongly depends on the time and severity of injury [4]. One particularly illuminating experiment conducted by Mocco et al. [20] showed that the deletion of C5 did not offer protection after transient focal ischemia (acute ischemic injury), but another group demonstrated a reduction in infarct size and attenuated neurological deficits in a model of permanent stroke associated with long-term activation of C5 [54]. Studies revealed that the action of C5a protects against glutamate-mediated neurotoxicity in mice [55,56]. In addition, a follow-up experiment showed that neuronal apoptosis induced by C5a under OGD conditions was prevented by its absence.

Finally, the functional role of MAC/C5b was also investigated. Deficiency of CD59, an inhibitor of MAC (a pore-forming complex that mediates cell lysis), increased infarct volumes, brain swelling, and greater neurological deficits in a model of mild stroke but not in a model of severe stroke in mice [57]. In summary, despite several inconsistencies, deficiencies of individual components of the classical pathway improve outcomes after adult brain ischemia.

In an effort to identify which activation pathway might also participate in complement system activity after brain ischemia, a few studies focused on the potential involvement of lectins and alternative pathways. However, the exact pathway has not been clarified, and this subject remains rather controversial. Pharmacological deficiency of MBL or neutralizing antibodies reduced infarct volume and neurological impairment [58,59,60]. Another set of data showed that there was no significant difference in infarct volume, brain oedema, or complement deposition between wild-type and MBL-deficient mice [61]. A more detailed study indicated that protection in MBL-deficient mice was pronounced only in the acute phase and not sustained in the subacute phase [62]. Thus, it is reasonable to speculate that the lectin pathway is insufficient to initiate complement activation. Moreover, inhibition of the alternative pathway through deletion of factor B in knockout mice and administration of CR2-factor H produced significant improvements in neurological scores and reduced ischemic volume and neutrophil infiltration [63]. These findings support the concept that the lectin and alternate pathways are significant contributors to the pathogenesis of stroke.

### 2.2. Neonatal Hypoxic-Ischemic Encephalopathy

Hypoxic-ischemic encephalopathy (HIE) in newborn infants remains one of the most important causes of death and/or long-term neurobehavioral and cognitive dysfunction. The development of injury to the neonatal brain is a complex process with multiple contributing mechanisms and pathways resulting in both early and delayed disturbances. Inflammation has long been implicated in the pathogenesis of HIE. In the past decade, new research focused on the functions of the complement system, an essential component of the inflammatory response.

Complement cascade proteins are widely expressed in the healthy developing brain and are responsible for synapse elimination, a key developmental process [64]. The abnormal synaptic morphology of neurons differentiated during the inflammatory state may lead to serious consequences with interruption of brain survival [65]. Thus, the complement system has assumed a prominent and unique role in processes associated with hypoxic-ischemic injury. Notable advances from clinical data showed reduced levels of the complement component C3 in the blood of neonates who subsequently developed cerebral palsy [66] and increased levels of peptides C3a and C5a after fetal acidosis [67]. In addition, a diminished mean C9 concentration in CSF samples was also detected. Moreover, the analysis of postmortem brain tissue 4–5 days after severe HIE revealed that activated C9 was deposited on neurons [68].

To evaluate the role of complement in neonatal HIE models further, two groups of investigators observed the effect of CVF treatment. However, the results obtained are inconclusive. One series of experiments revealed that CVF diminished post-ischemic cerebral infarct volume and atrophy in neonatal rats and decreased C3 component deposition [25]. In contrast, two years later, Figueroa et al. [26] did not observe a beneficial effect from administration of this factor. A main point of confusion is that investigators applied different experimental paradigms. These studies demonstrated increased expression of genes related to complement activation [69,70]. Insight into the mechanisms mediating inflammatory neonatal brain damage after HI came from experimental studies showing the deposition of C1q and C3 and the generation of proinflammatory mediators such as C5a [22,71,72].

A more precise study performed by Ten [73] revealed that deletion of C1q in mice conferred significant and long-lasting neuroprotection as expressed by diminished brain infarction and improvement of functional impairments compared to wild-type controls. The same group showed that neurons of C1q−/− mice are resistant to HI with preservation of brain mitochondrial respiration and reduced production of reactive oxygen species. The results highlight the importance of the classical C1q-dependent activation pathway after HI.

It is worth noting that in neonatal mice, unlike in adult rodents, the nonclassical pathway is underdeveloped. In this case, the genetic deletion of C1q results in inhibition of the terminal C cascade response to HI and is sufficient to confer neuroprotective effects. Indeed, decreased deposition of C3 in the infarcted brain coupled with a lesser degree of brain damage in C1q−/− mice compared with WT mice suggests that C3 activation plays an important role in the exacerbation of brain damage. However, the findings of Jarlestedt et al. [74] provide evidence that the C3a peptide is neuroprotective against HI-induced injury to the immature brain. It ameliorates memory impairment after neonatal HI. The beneficial effect of C3a was also supported by results published by Moran et al. [75]. The data described above are consistent with the suggestion that increased interactions of C3a–C3aR during hypothermia induced in hypoxic neonates contribute to decreased inflammation and tissue damage [72]. The beneficial effect of C3a was supported strongly by results described by Moran et al. [75]. They found that intranasal treatment with C3a demonstrated neuroprotective action against HI. In addition, this approach ameliorated injury and induced reactive gliosis in the hippocampus but was not able to reduce the extent of hippocampal tissue loss.

The last component to be studied was proinflammatory C5a, generated by classical complement pathway activation. The elevation of C5a demonstrates deleterious consequences in the early phase. The function of C5a is mediated by its receptor C5aR, which is expressed predominantly in microglia. Mice with C5aR deletion presented only short- but not long-term improvement. In addition, therapeutic hypothermia combined with inhibition of the expression of C5aR1 was able to reduce brain infarct within three days of brain ischemia [72]. The effects in the later stages were not yet explored.

Analysis of the C9 component, a marker for C5b-9 assembly, showed that deposition of C9 in neurons in the infant brain appeared to be neurotoxic [76]. Thus, C9 deficiency reduced the brain infarct volume, whereas the reverse action was induced after C9 administration [77].

In summary, the results presented illustrate the different roles of complement proteins in ischemic injury between immature and adult brains. Therefore, the findings obtained in adults cannot be extrapolated to HI-induced injury in the immature brain.

### 2.3. Traumatic Brain Injury

Traumatic brain injury (TBI) is a consequence of an external mechanical force that induces disruption of the normal structure and function of the brain tissue and blood vessels. Pathophysiology following TBI is classified into the primary (initial) injury and secondary injury that develops subsequently, a determinant of outcomes. Secondary injury is generally associated with a cascade of pathophysiological processes, including glutamatergic excitotoxicity, calcium overload, and neuroinflammation, which are thought to be some of the leading factors inducing damage [1,78,79].

Increasing evidence indicates the involvement of complement-driven networks in inflammatory processes; thus, the complement system is thought to play a particularly significant role in the subsequent injury that occurs in the context of TBI. One piece of the evidence collected showed increased immunoreactivity for certain complement components (C1q, C9, C3 C5b-9, MAC, and FB) in CSF, blood plasma, and in the immediate vicinity of neurons in the penumbra of TBI patients [80,81,82,83,84].

Evidence for the impact of the complement in TBI neuropathology come from a variety of experience-based animal models (cryoinjury, controlled cortical impact, or weight drop). The results, despite several inconsistencies, indicate that certain alterations of complement proteins could improve outcomes after TBI. Early studies by Kaczorowski et al. [85] revealed that suppression of C3 convertase formation by administration of the soluble complement receptor sCR1 reduced neutrophil filtration [85]. After this discovery, it became clear that the complement plays a pivotal role in the neuroinflammatory response. The beneficial role of the inhibition of C3 convertase formation was further confirmed by C3 deletion and/or C3 systemic inhibition via administration of recombinant chimeric Crry-Ig. Both procedures resulted in reduction of microglial activation/infiltration, improved cognitive and functional recovery, and diminished extent of neuronal cell death. Likewise, decreased neuronal death post-TBI was also noted after targeted deletion of the *cfb* gene, which stimulates C3 convertase [86,87]. Altogether, a beneficial effect of C3 inhibition could be proposed as an attractive drug target [88,89,90,91,92,93].

Other research groups working at the same time demonstrated the participation of two components, C4 and C5, in the pathogenesis of TBI, as deficiency of these components and the presence of their antagonists reduced secondary damage in some models of TBI [89,90,92]. Within a few years, additional work performed by Stahel et al. [94] indicated the detrimental role of the terminal complement pathway, MAC (C5b-9). They found that overexuberant MAC formation is an important predominant factor implicated in secondary injury following TBI. Consistent with this hypothesis is the beneficial effect of the complement inhibitor OmCI, which binds C5 and blocks MAC formation. This paradigm decreased neuropathology and protected recovery [95]. A similar neuroprotective effect was noticed by another inhibitor of MAC formation, the CD59-CR1 hybrid (which localized to areas of C3b/iCb deposition in the injured brain) [96]. Therefore, it is concluded that the final pathway may function as a therapeutic target because its inhibition prevents the amplification of C3 and C5 convertase generation required for MAC formation.

One further notable advance is the recognition of the important role of factor B. The targeted deletion of the factor B gene extended the survival of neuronal cells in mice. This response may indicate the importance of the alternative complement pathway in the pathophysiology of TBI [86]. Thus, site-targeted alternative pathways may represent a novel therapeutic avenue [91].

Finally, by analyzing the lectin-dependent pathway, surprisingly, the pathogenic role of MBL was identified. However, data reported by different groups are truly conflicting. According to De Blasio et al. [97] and Longhi et al. [98], inactivation of the lectin pathway using a multivalent MBL ligand improved functional and pathological outcomes and decreased cortical cell death in mouse TBI. In contrast, MBL deficiency increased the number of degenerating neurons and exacerbated neurological disturbances [99]. In addition, one study showed that there is no correlation between lectin complement pathway activation and mortality/consciousness after severe TBI [100]. These conflicting findings may be related to different experimental paradigms (severity of TBI and time analysis). It is postulated that this pathway may play a dual role. The specific neuroprotective capacity may be demonstrated in the early phase of TBI secondary injury before switching to a deleterious phenotype in the late phase.

### 2.4. Spinal Cord Injury

Spinal cord injury (SCI) is caused by sudden traumatic insult damaging neural tissue. It results in dysfunction and sometimes loss of function below the lesion sites. Several mechanisms may contribute to secondary pathology caused by SCI, including axonal injury, demyelination, excitotoxicity, oxidative damage, and inflammation frequently associated with disruption of the blood-spinal cord barrier and recruitment of immune cells [101]. It was assumed that complement activation plays an important role in the inflammatory response [102]. The involvement of components is described in detail in a review published within the last year by Lee et al. [11]. Therefore, we will only briefly report the current data.

The detection of elevated levels of certain components, C3, C4, and C5, in the plasma of patients post-SCI provided insightful views on their role in pathology [103,104]. Furthermore, analysis performed in several animal models of SCI showed that complement proteins, including C1q, C4, FB, C3, MAC-C5b9, and complement regulator factor H, were deposited in neurons and oligodendrocytes at injured sites [105,106,107,108]. In addition, the ability to visualize C1q and factor B in axons provided views into the role of complement activation in demyelination or axonal degeneration [105]. The participation of the complement system in SCI was confirmed using knockout mice. In fact, C1q-, FB-, C9-, and C3-deficient animals exhibited reduced lesion sites at the injury epicenters, reduced infiltration of neutrophils and macrophages, and, to some degree, improved functional recovery. Moreover, administration of the complement inhibitor CR2-Crry or a factor B antibody also improved neurological deficits [109,110,111]. It is worth noting that the injured spinal cord in SCI is vulnerable to complement overactivation, for example, in the absence of the complement regulator CD59. The lack of regulators exacerbates neuropathology in SCI and increases MAC-C5b-9 deposition [111].

Recent findings indicate a more complex function for the complement cascade. For example, the role of C5aR1 evolves with time after SCI. The initial pathogenic role demonstrated a later delayed neuroprotection [11,112]. In addition, Lee et al. [11] revealed the protective function of other complement receptors, C5aR2 and C3aR. However, until now, data relating to the pathology of SCI were insufficient, and there are no proven therapies. There have been only a few studies using anti-complement treatment. However, the timing of intervention may be crucial to avoid impacting the deleterious effect of the complement.

## 3. Neurodegenerative Diseases

Neurodegenerative diseases constitute a heterogeneous group of disorders that increase in incidence as the population ages. These disorders are characterized by a progressive decline in cognitive ability and memory formation, leading to profound dementia. A common feature of these diseases is neuroinflammation, and it is logical to suggest that complement components contribute to pathogenesis [102]. Indeed, several studies showed that dysregulated systems and excessive complement-mediated synapse loss are associated with neurological disturbances. The most common diseases include AD, PD, HD, ALS, and MS. In this review, we do not consider AD because this was recently discussed elsewhere [113,114]. We present the key findings for complement participation in selected neurodegenerative diseases.

### 3.1. Parkinson Disease

Parkinson disease (PD) is a progressive, degenerative disease that leads to the loss of dopaminergic neurons in the substantia nigra (SN), causing depletion of dopamine in striatal projections [115,116]. The main feature of this pathology is progressive motor dysfunction. The neuropathological hallmarks required for a PD diagnosis are intracellular protein aggregates called Lewy bodies, consisting predominantly of alpha-synuclein and ubiquitin. Although the mechanism that triggers brain degeneration in PD is unknown, several factors (such as altered mitochondrial activity, loss of trophic factors, abnormal kinase activity, proteosomal and lysosomal dysfunction, and neuroinflammation) are thought to be the key components in the pathogenesis of disease [117,118]. In recent decades, the complement system received considerable attention as an important mediator of inflammatory responses in several neurological disorders. Hence, the possible linkage of the complement with PD was postulated. The first observations of activated complement products C3d and C4d within the postmortem brains of PD patients were reported in 1980 by McGeer and McGeer [119]. A few years later, the same group of researchers found a marked elevation in the mRNA levels of complement components in regions affected by PD [120]. Then, a subsequent series of publications identified several components—C1q, C3d, C4d, C7, and C9—in neuronal Lewy bodies of PD patients [121,122,123]. In addition, alteration of complement factors within the blood of PD patients was also detected [124]. Further systemic in vitro studies showed that activation of the complement is caused by the disease-associated splice variant of alpha-synuclein-112 but not the full-length protein [125]. In the same year, Wang et al. [126] demonstrated that C5a synergized with IgG isolated from the serum of PD patients led to selective death of dopaminergic neurons in rat mesencephalic neuron-glia cultures. Nevertheless, despite the continued insufficiency of data, it is postulated that the complement system is involved in PD pathology. Furthermore, the exact functional roles of complement components in this disorder are not yet clarified.

The absence of C1q and C3 did not protect against the depletion of dopaminergic neurons in a toxin-induced MPTP mouse model [121,127]. The beneficial effect expressed by the protection of dopaminergic neuron loss and motor dysfunction was observed after deletion of receptor CR3 in mice. This observation clearly suggests that CR3 contributes to the disease process [128]. The mode of CR3 action is associated with the activation of microglial NADPH oxidase and subsequent degeneration in a toxin-induced PD mouse model. Finally, the relevance of these observations to PD pathology remains notable. However, the available data are largely insufficient, in contrast to information on other CNS diseases, and do not allow us to define the role of the complement system in PD.

### 3.2. Amyotrophic Lateral Sclerosis

Amyotrophic lateral sclerosis (ALS) is a fatal neurodegenerative disease associated with progressive degeneration of upper and lower motor neurons. Damage to motor neurons and denervation of neuromuscular synapses in the peripheral nervous system result in the loss of control of voluntary muscle movements, spasticity, respiratory failure, and ultimately paralysis and death within 2–5 years of diagnosis [129,130]. Although the exact mechanism precipitating motor neuronal death is not yet precisely defined, subsequent analyses of animal models and human patients identified a plethora of pathological events in the course of ALS, including increased glutamate-mediated excitotoxicity, oxidative stress, mitochondrial dysfunction, and sustained upregulated immune responses, accompanying the pathogenesis of ALS [131,132]. However, the primary process leading to ALS pathogenicity remains a matter of discussion. New evidence indicates the contribution of complement-driven mechanisms in the promotion and development of both familial and sporadic forms of the disease [114,133]. The increased expression of upstream complement components C1q, C3, and C4 in the peripheral blood and spinal cerebral fluid (SCF) in living ALS patients and in the postmortem motor cortex and spinal cord in proximity to motor neurons found in ALS individuals suggests an ongoing complement activation process [134,135,136,137]. In addition, the increased deposition of the terminal component MAC/C5b-9 in motor end-plates in muscle biopsies from ALS patients further supports the engagement of the system in the disease. Moreover, it was hypothesized that the complement components may affect outcomes [102,138,139,140]. Another set of supporting information came from examining animal models, which recapitulated the onset and progression of ALS-hSOD1^G93A^ and TDP43^Q31k^. These studies have revealed increased expression of complement genes and proteins C1q, C4, C3, C5, and factor B detected early in the spinal cord and skeletal muscle in the disease process in transgenic mice overexpressing mutant SOD1 [134,141,142,143,144,145,146,147,148]. The detailed study of Bahia et al. [134] showed deposition of C1q and C3/C3b at the motor end-plate before neurological symptoms. Notably, complement deposition contributes to nerve terminal destruction in ALS [149]. The described alteration of the complement was closely linked to the reduced expression of the regulatory proteins CD55 and CD59a, regulating C3 and MAC, respectively [144,145,147]. The next dataset published by these authors suggested that complement dysregulation could play a role in motor neuron loss and denervation of neuromuscular junctions.

Considering these results, it seemed surprising that, despite the upregulation of C1q, C3, and C4 components, genetic deletion of the upregulated components failed to prevent or at least delayed the onset of disease in hSOD1^G93A^ transgenic mice [149,150]. Importantly, deletion of the gene encoding C4 significantly reduced numbers of activated macrophages found in the sciatic nerves of mSOD1^G93A^ mice but, despite this effect, also failed to influence disease course. Therefore, all the data described above provoked the suggestion that the complement does not contribute to ALS progression.

This proposal contrasted somewhat with results showing that genetic deletion or pharmacologic blocking of the downstream C5aR1 with antagonist PMX205 ameliorated motor deficits and extended survival in mutant mouse models (hSOD1^G43A^, TDP-43^Q331K^) [144,148,151]. Thus, it was concluded that C5a-C5aR1 signaling may affect disease progression through the activation of local immune cells and infiltration by macrophages, leading to an overall increase in inflammation/neuroinflammation and then neurodegeneration [147,148,152,153]. The contribution of strengthened C5aR1 signaling to motor neuron death was further supported by studying cell cultures [136,154].

The reasons for the conflicting results among C1q, C3, and C4 knockout, indicating lack of effect and beneficial C5aR1 deletion/antagonism, currently remain unclear, but it seems logical to suppose that in the absence of upstream complement proteins, there is downstream pathway compensation, potentially allowing for pathological terminal complement activation and C5a/C5AR1 engagement to occur [153].

In view of the suggested prominent pathogenic involvement in ALS, complete understanding of the mechanism by which C5aR1 contributes to ALS may lead to optimal target selection for all forms of ALS, depending on the stages and severity of disease.

### 3.3. Huntington Disease

Huntington disease (HD) is a dominantly inherited autosomal disorder caused by expansion of three-base-pair (CAG) repeats. The CAG repeat is translated into an expanded polyglutamine tract in the ubiquitously expressed huntingtin protein within neurons and glial cells [155]. The inclusion of huntingtin leads to cognitive defects, motor dysfunction, psychiatric impairments, and subsequent neuronal loss [156]. The degenerative process is most severe in the striatum and cerebral cortex but is ultimately observed throughout the brain. However, how trinucleotide expansion in the huntingtin protein is linked to the observed disorders remains unclear. More recent studies implicate the immune system as being associated with HD pathology [157,158,159]. Because complement cascade activation is the best-known feature of the immune response, the detailed characterization of component functions in HD has become crucial due to the established strong association of the complement system with common diseases. One notable advance in this research came from the discovery of complement activation products in postmortem brain tissue from HD subjects. Furthermore, the expression of mRNA coding early components (C1q, C1r, C4, C3), complement regulators, and membrane cofactor proteins (CD46, CD55, and CD59) was upregulated in the striatum of HD patients [50]. The activation of the complement was further supported by findings of Hodges et al. [160], who observed increased expression of C4A, C4B, and C3 in the caudate nucleus and motor cortex. Additionally, some reports showed the elevation of certain components (C3, C4, C7, and C9) in the cerebrospinal fluid, serum, and plasma from HD patients. Importantly, detailed analysis of HD individuals revealed that the concentrations of these factors are positively correlated with disease severity [158,161]. It is worth noting that elevated levels of C4 and C7 were detected even before visible symptoms, while the level of C9 remained unchanged until an advanced stage of disease [158].

Taken together, the above data suggest that dysregulation of the complement system contributes to HD pathogenesis. This hypothesis was confirmed by using experimental models of HD. Significantly elevated levels of C3, C9, C5aR1, and C5aR2 were observed in the striatum of rats after administration of 3-nitropropionic acid (3-NP), which caused striatal degeneration [7]. Remarkable improvement in general knowledge relating to the role of the complement came from the beneficial effect of the receptor C5aR1 inhibitors PMX53 and PMX205. This approach reduced 3-NP-induced neuronal death and gliosis and ameliorated disease pathology and behavioral deficits in transgenic mice (R6/2 and C57BL/6). The relevance of C5a-C5aR1 to HD remains to be determined. Surprisingly, deletion of C3, the upstream component of the complement cascade, in a more relevant transgenic model of HD (R6/2) did not change disease progression and did not influence disease pathology [162]. Similarly, the lack of such an effect seems to be more general, as it was also observed in ALS [150] and PD models [127].

### 3.4. Multiple Sclerosis

Multiple sclerosis (MS) is a complex autoimmune demyelinating disease with a variable pathology and phenotypic presentation and an unknown disease course that cannot be predicted. The disease is characterized by recurrent episodes of inflammatory demyelination with a relapsing-remitting course, significant synapse loss, and CNS atrophy [163,164,165]. Although the exact molecular basis responsible for the pathogenesis of MS is still not clearly understood, increasing evidence suggests an autoimmune disorder that results in multiple inflammatory processes. Therefore, it seems logical to anticipate the participation of complement molecule activity in MS, similar to observations in other neurodegenerative diseases.

Growing clinical data show higher levels of various complement compounds in the plasma and CSF of MS patients than in healthy controls. Moreover, initial inspection of the database revealed that a large majority of increased component expression is closely related to the severity of disease [166,167,168]. Therefore, components C3 and C4a were significantly elevated in the serum of patients in the active relapsing-remitting (RRMS) phase compared to the stable stage of MS [166,167,169]. Similarly, enhanced expression of C3, C4b, and terminal complex C5b-9 was found in CSF in the matching phase of the disease [169,170]. Further research revealed that elevation of complement regulatory protein factor F can predict the transition from relapsing to progressive disease, since factor H increased progressively with disease advancement in patients transitioning from RRMs to secondary MS progression [167]. Another study detected an increase in soluble complement receptor 2 (sCR2) in patients with either RRMS or SPMS compared to controls [171].

It is worth noting that only a few studies were performed to clarify the involvement of the mannose binding lectin (MBL) pathway. However, despite the scarce data, a positive correlation between plasma levels and severity scores was demonstrated [172,173]. It is believed that full activation of the complement cascade may be restricted to patients with more advanced disease and is significantly correlated with the degree of neurological disability. Thus, according to existing knowledge, it is tempting to speculate that the presence of complement components and activation products in serum and spinal cortical fluid may serve as biomarkers of activity and probably help to differentiate the various MS subtypes [174].

The upregulation of complement components C1q, C3b, C4d, C3aR, and C5aR C5b-9 (MAC) was also evident in postmortem human tissue and in preclinical models of MS, suggesting the involvement of classical and alternative pathways [175,176,177]. The complement proteins were localized predominantly within plaques and adjacent white matter areas, in microglia, in synaptic elements, and in capillary endothelial cells. Information on all C activation products is consistent with possible direct involvement in myelin degradation in MS patients by lysis of oligodendrocytes and chemoattraction of macrophages to the inflammation sites [178]. For example, direct evidence for the role of MAC, the final complement product, in inflammatory disease progression in a mouse EAE model was documented by the effect of an inhibitor that prevents relapse after the first clinical attack. In addition, inhibition of the C5aR1 receptor reduced inflammatory gene expression [179]. This finding provides strong support for conclusions of earlier reports indicating that MAC exerts a direct impact on nervous tissue destruction in MS [175,177]. It should be pointed out that human myelin vulnerability to complement attack may be caused by the lack of complement inhibitors [180].

A component of MS pathology, in addition to demyelination and axon degeneration, is synapse loss. In this context, the compounds that attracted a great deal of attention are C1q and C3. The data reported by Michailidou et al. [179] in an EAE mouse model indicate that the main role in synapse loss is played by axis C1q-C3. Robust deposition of both complement components at synapses and colocalization with the receptor CR3 mediate signals that could strengthen synapse vulnerability to phagocytosis by microglia [181,182]. This statement is consistent with findings of earlier work showing that mice deficient in CR3 display an approximately 50% reduction in microglial synaptic engulfment in a model of AD [183,184]. Subsequent examination of synaptic changes in demyelinating disease was performed by Werneburg et al. [185]. The results obtained demonstrated the prominent role of C3 in synapse elimination. Moreover, they indicated that this process occurs through activation of the alternative complement pathway, contrary to the classical pathway engagement postulated by Michailidou et al. [182]. Thus, it is postulated that the elevated level of C1q may drive inflammatory reactive gliosis concomitant with the loss of synapses. The function of C3 was also confirmed by using the specific C3 inhibitor AAV-Crry. The presence of this agent leads to decreased microglial engulfment of synapses and preserved circuit function. This may indicate a strategy to prevent synapse loss. In addition, genetic deletion of C3, but not knockout of C1q, significantly reduced the EAE clinical score and preserved hippocampal synaptic density [186]. Another set of informative data regarding the participation of alternative pathways in MS came both from analysis of genetic deletion of factor B and the use of specific anti-B antibody. Both treatments showed promising results with delayed onset and reduced severity of EAE symptoms [187,188].

Although alternative pathway activation of the complement system in MS was documented, there remains a great deal of uncertainty regarding the roles of other pathways in this disease [189].

### 3.5. Epilepsy

Epilepsy is a chronic neurological disorder characterized by an enduring predisposition to recurrent spontaneous and unpredictable seizures. The clinical manifestation of epilepsy includes sudden and abnormal episodes of motor, sensory, autonomic, or psychological origin. Comprehensive studies revealed that epilepsies include a broad range of central nervous system disorders with different, not yet clarified, complex behavior at the molecular and cellular levels. A body of collected evidence demonstrated that inflammatory and immune processes are possible mechanisms controlling seizure recurrence and precipitation in both epileptic patients and animal models [190,191,192,193,194,195]. Noting the data indicating that the complement system is a major component of the innate immune system and participates in adaptive immunity, it seems logical to suggest its potential link to epileptic conditions. However, only a few studies showed the role of the complement system in the pathogenesis of epilepsy [196,197,198]. As a result, increased expression of genes involved in the complement pathway following seizures was reported and found to remain elevated during the chronic phase in a rat model of temporal lobe epilepsy (TLE) [199,200]. Furthermore, concentrations of multiple complement components, C1q, C3, C4, and the MAC consisting of the C5b-C9 complex, were documented to be higher than in healthy controls in surgically resected brain samples collected from patients and in experimental animal models of TLE epilepsy [190,199,201,202,203,204]. Epilepsy-induced increases in C1q signaling and the generation of C3a- and C3b-mediated activation of the C5a/band may contribute to the initiation and/or preservation of neuroinflammation in epilepsy [205]. This finding parallels altered expression of inflammatory cytokines that are widely associated with generation of seizures and epilepsy [192,206]. Moreover, the induction of both behavioral and electrophysiological seizures and neuronal death were also described after sequential infusion of individual MAC proteins (C5b, C7, C8, and C9) into the hippocampus [207].

Conversely, a follow-up study surprisingly revealed that complement component factor H (CFH), a specific inhibitor of the C3 to C3b transition, was downregulated in rat hippocampal tissue after epilepsy induced by electrical stimulation. The downregulation promoted acute seizures and upregulation of reduced seizure susceptibility. This suggests that CFH may contribute to epileptogenesis [208].

The contribution of complement components to epilepsy was supported strongly by experimentally blocking the C5aR1 receptor with a PMX53 antagonist. Such an approach diminished seizure power and protected hippocampal neurons from degeneration and epilepsy-associated mortality in two mouse models of epilepsy (induced by pilocarpine and intrahippocampal kainate) [209]. Moreover, C5aR1 deficiency was accompanied by reduced inflammation expressed by attenuation of TNF-alpha upregulation by microglia. This finding may represent an important opportunity for the prevention of epileptogenesis.

Furthermore, in another experimental setting, a C1q inhibitor blocked the classical and lectin pathways in rats following pilocarpine-induced epilepsy and showed at least some neuroprotective effects, including microgliosis promotion and accelerated weight gain, but had no effect on memory deficits [210]. It was postulated that the classical complement pathways C1q and C3b are involved in epileptogenic remodeling of the synaptic course and limit synaptic connectivity [204,211,212,213]. This suggestion is consistent with the reported removal of synapses during development of the visual system and the elimination of unnecessary structures of the synaptic hippocampus in models of neurodegeneration [64,184,214,215]. However, until now, there was a lack of direct data about the possible role of the complement in epilepsy processes. This subject will require additional investigation.

## 4. Psychiatric Disorders

Accumulating evidence suggests a dysregulated complement pathway involved in the pathogenic processes of psychiatric disorders such as schizophrenia (SZ) [216] and autism spectrum disorder (ASD) [217]. Abnormal complement signaling owing to genetic mutations or as a result of inflammatory insult during pre- and postnatal development may lead to changes in brain connectivity and may contribute to disease pathophysiology.

The present section summarizes the current data related to the role of the complement system in selected psychiatric disturbances—schizophrenia and autism spectrum disorder.

### 4.1. Schizophrenia

Schizophrenia (SZ) is a chronic and disabling mental disorder marked by symptoms including psychosis and deficits in cognition and social interactions. SZ commonly develops during late adolescence or early adulthood [218]. The principal pathologic findings in the brains of those affected with SZ include excessive loss of grey matter and fewer dendritic spines on neurons from the prefrontal cortex [219,220,221]. An early hypothesis stated that SZ may result from faulty synapse elimination in the postnatal period [222]. To date, the exact mechanism underlying schizophrenia is not clear. Studies in recent decades uncovered complex interactions among the immune system, systemic inflammation, and disturbed brain function demonstrated by changes in mood, cognition, and behavior [223,224]. Since then, elevated levels of inflammation markers (TNF-alpha, IL1-beta, IL6) in peripheral blood largely confirmed the participation of inflammation in SZ, at least in a subset of individuals with SZ [225,226]. Considering the established role of the complement as a mediator of innate and acquired immunity and its role in synapse elimination (as in neurodegenerative diseases), it is not surprising that alterations in complement genetics, or complement expression, might be linked to SZ. Regarding this matter, a series of studies reported changes in the hemolytic activity of classical pathway components, particularly C1q but also C2, C3, and C4, in SZ patients. The results obtained were inconsistent, and often failed to be replicated and have not provided significant insights into pathogenesis [227,228,229,230,231,232]. Nevertheless, these findings pointed towards abnormal activation of the classical complement pathway; however, the function of several components in SZ are not yet clarified. An example is C1q, whose increased level in the sera of schizophrenia patients, compared to those of controls, may be involved in linking autoimmune processes [223]. Additionally, elevated levels of C1q in blood cells were detected in mothers of infants who later developed schizophrenia. Therefore, it is postulated that C1q may be a contributing factor to the disease [233].

The availability of the large-scale genome-wide (GWAS) methodology provided clear views into the association between the complement system and schizophrenia. Through a collaborative effort of the Schizophrenia Working Group of the Psychiatric Genomics Consortium [234] and International Schizophrenia Consortium [235],^,^ it was discovered that the locus most significantly associated with SZ lies within the extended human major histocompatibility complex (MHC), and the genomic region lies near the region encoding complement component C4. However, the precise determination of which genes force the SZ-MHC association is difficult due to extensive polymorphism. The subsequent research of the Schizophrenia Working Group of the Psychiatric Genomics Consortium [234] and Schizophrenia Psychiatric Genome-Wide Association Study Consortium [236] identified the connection of schizophrenia to *CSMD1,* which encodes a gene product that regulates the complement system by degrading C4 and C3. Deficiency in this factor is characteristic of SZ symptoms, such as diminished cognitive ability and memory function [237,238,239]. Thus, it was speculated that the association of SZ with *CSMDI* may indicate that C4 is a convincing genetic marker of schizophrenia [216].

The C4 component is encoded by two different genes, C4A and C4B, exhibiting distinct relationships with schizophrenia risk. The analysis revealed a strong correlation between schizophrenia risk alleles and copy number variation in the C4A gene. Additionally, postmortem analysis of brains from individuals with SZ showed higher expression of C4A than that in controls. It was therefore concluded that the presence of the C4A gene and high C4A expression predispose patients to SZ [216]. Subsequent brain imaging studies using magnetic resonance spectroscopy confirmed the association between high C4A gene copy numbers and neuropil contraction in different brain regions of patients with SZ [240].

The association of the C4A gene with schizophrenia and the presence of the C4 protein in synapses lead to the speculation that the pathogenic effect of this component may be mediated through dysfunction of synapse elimination and thus loss of important aspects of brain connectivity and sociability, consistent with the early hypothesis of Feniberg [222]. Recent work suggested that C4 might work with other components of the classical cascade to promote synaptic pruning by microglia. According to a report by Ishii et al. [241], the altered level of C5 may be associated with decreased cortical thickness [241,242].

It is worth noting that from the initial identification of C4A as a risk factor in SZ, several reviews on this subject were published [243,244]. Data from the literature point rather towards increased classical pathway involvement compared to controls. Data regarding alternative or lectin pathways [231] are too scarce to draw any conclusions. Similarly, the data related to the treatment of schizophrenia regarding the complement system are limited for the reasons presented in the elegant and detailed review by Woo et al. [244].

### 4.2. Autism Spectrum Disorder

Autism spectrum disorder (ASD) comprises a heterogeneous group of early-onset neurodevelopmental large-scale neuronal network diseases. Autism is characterized by impaired social communicative cognition, language impairment, and restricted and repetitive types of behavior [245,246]. Despite the considerable speculation about causes of ASD (such as neurotransmitters, genetics, environmental factors, and many others) [246,247,248], the exact pathophysiology of ASD remains unknown. However, the evidence presented thus far strongly suggests a potential role of immune activation/inflammation as a risk factor contributing to this disorder [249,250,251,252]. In the past decade, research emerged providing insight into the potential role of the complement system, the major effector of immunity, in ASD. One of the notable findings came early from Warren et al. [253], who described increased frequencies of C4B alleles in autistic patients and their mothers. Further breakthroughs in research on the participation of the complement system in ASD were presented in a series of publications reporting altered levels of complement molecules in the periphery of subjects with ASD. For example, increases in levels of C1q and C3 and C3 fragments were found in the plasma of children with this disorder [254,255]. In addition, analysis of postmortem brain tissue from ASD patients detected increased dendritic spine density, probably resulting from the reduction of synaptic pruning associated with the activation of the complement system [256,257].

Direct evidence on the association between complement components in the brain and ASD was reported by Fagan et al. [217]. Analysis of human postmortem samples from ASD patients showed elevated levels of mRNA coding for C2, C5, and MASP1 (MBL-associated serine protease 1) and decreased C1q, C3, and C4 mRNA levels in the middle frontal gyrus of ASD subjects. However, the availability of brain samples was not sufficient to make a confident conclusion. He further checked the effect of C3 knockdown in the prefrontal cortex (PFC) in rodents. The deletion of C3 resulted in social deficits and repetitive behavior in mice. This may indicate a possible role of C3 in the pathophysiology of ASD. Therefore, it is tempting to speculate that diminished complement-mediated synaptic pruning, among other mechanisms, may contribute to cortical hyperconnectivity and behavioral phenotypes in ASD. This is consistent with the previous statement that C3 deficiency may limit synaptic pruning processes [184].

Summarizing the available data, the evidence regarding the association between complement dysfunction and ASD is far weaker than the evidence of complement dysregulation in schizophrenia susceptibility. In addition, a recent genome-wide association did not identify common variants in complement genes significantly associated with ASD [258]. However, the altered complement expression in peripheral blood and in the brain from patients might suggest that ASD may somehow be attributed to aberrant activity.

## 5. Conclusions

Our review outlines recent discoveries that indicated complement system involvement in a wide range of brain disorders contributing to neuronal and synapse remodeling (summarized in Table 1). Over the last decade, understanding of the participation of complement components in brain pathology greatly advanced; however, the molecular mechanisms leading from alteration of the system to disturbances in brain functionality are not fully understood. The examination of the responses of different components to pathological conditions proved to be somewhat controversial, probably due to differing experimental paradigms. In addition, in many cases, the reported results are rather scarce and do not allow us to determine the roles of particular factors precisely. However, despite certain inconsistencies, dysregulation of the complement system pathways affords either beneficial or detrimental effects, depending on the stage of disease. It is hypothesized that modulation of specific members of the pathway could serve as an effective therapeutic approach for attenuating or ameliorating disease-specific signs. Therefore, future research should be focused on advancing the precise understanding of these processes.

## Author Contributions

T.Z. was the major contributor in collecting data and writing the manuscript. M.Z.-N., K.Z., P.P., and J.S. participated in writing the manuscript and prepared figures. All authors have read and agreed to the published version of the manuscript.

## Figures and Tables

**Table 1:** The effects of dysregulated complement system in neurological disorders in humans and animal experimental models.

	Humans/Patients	Animal Experimental Models
Brain ischeamia	increased expression of several complement components: C3, C3a, C5b-9 in plasma/serum [18,19,20,21,22,23] deposition of C1q, C4d, C3c, C9, and MAC/C5 in the ischemic regions of brains (post-mortem) [15,19,20,21,22]	inhibition/deletion of C3 and C3aR presents protective action by improving outcome [39,40,41], but impairs ischemia-induced neurogenesis suppressing repair [12,42,43,44] protective effect of C5a depends on the time and severity of injury [54,55,56] deficiency of CD59, an inhibitor of MAC, presents deleterious effect (increased infarct, brain swelling, and greater neurological deficits) [57] inhibition of MBL presents beneficial effect in the acute phase (reduces infarct volume and neurological impairment) [58,59,60,62] inhibition of the alternative pathway (deletion of factor B) shows neuroprotection [63].
Neonatal HIE	decreased expression of C3 in the blood of neonates [66] increased levels of C3a and C5a [67] diminished C9 concentration in CSF and activation C9 in neurons (post-mortem) [68]	deficiency of C1q is neuroprotective (diminishes brain infarction and improves functional impairments). It is due to preservation of brain mitochondrial respiration and reduced production of reactive oxygen species [73] C3a ameliorates memory impairment [74,75] increased interactions of C3a–C3aR during hypothermia contribute to decreased inflammation and tissue damage [72] C5aR deletion resulted in short-term improvement [72] C9 deficiency reduced the brain infarct volume after HI [77]
Traumatic brain injury	increased expression of several components: C1q, C3, C5b-9, C9, MAC, and FB in CSF, blood plasma, and in the penumbra of TBI patients [80,81,82,83,84]	deletion and/or inhibition of C3 as well as the deficiency of factor B diminished neuronal cell death, improved cognitive and functional recovery [85,86,87] deficiency of C4 and C5 reduced secondary damage [89,90,92] inhibition of MAC formation decreased neuropathology and protected recovery [95,96] the role of lectin dependent pathway is conflicting. Whereas MBL ligand improved functional and pathological outcomes [97,98], MBL deficiency increased the number of degenerating neurons and exacerbated neurological disturbances [99]
Spinal cord injury	increased expression of C3, C4, and C5 in the plasma of patients post-SCI [103,104]	C1q, C3, C4, MAC-C5b9, FB, and factor H were deposited in neurons and oligodendrocytes at injured sites [105,106,107,108] deficiency of C1q, C3, C9, FB diminished lesion sites, reduced infiltration of neutrophils and macrophages, and improved functional recovery [109,110,111] the role of C5aR1 evolves with time after SCI (pathogenic at the early phase after the insult and neuroprotective at the longer time) [112] C5aR2 and C3aR have protective functions [12]
Parkinson disease	increased expression of C1q, C3b, C9-in neuronal Lewy bodies [121,122]	deletion of CR3R has the beneficial effect expressed by the protection of dopaminergic neurons loss and motor dysfunction [128] the deficiency of C1q and C3 did not show the protection effect [121,127]
Amyotrophic lateral sclerosis	increased expression of C1q, C3, and C4 in the peripheral blood and spinal cerebral fluid in living ALS patients and in the postmortem motor cortex and spinal cord [134,135] increased deposition MAC/C5b-9 in motor end-plates in muscle biopsies [136]	increased expression of C1q, C4, C3, C5, and factor B, in spinal cord and skeletal muscle in transgenic mice overexpressing SOD1 [134,141,142,143,144,145,146,147,148] genetic deletion of C1q, C3, and C4 failed to prevent or at least delayed the onset of disease in hSOD1^G93A^ transgenic mice [149,150] deletion/inhibition of C5aR1 ameliorated motor deficits and extended survival of hSOD1^G43A^ and TDP-43^Q331K^ mice [144,148,151]
Huntington disease	upregulated expression of C1q, C1r, C4, C3 in the postmortem striatum [50,160] elevation of C3, C4, C7, and C9 in the cerebrospinal fluid, serum, and plasma [158,161]	increased level of C3, C9, C5aR1, and C5aR2 in the striatum of rats after 3-NP administration [7] deletion of C3 in transgenic model of HD (R6/2) did not change disease progression [162] deficiency of C5aR1 presented beneficial effect expressed by amelioration of disease and behavioral deficit [7]
Multiple sclerosis	elevation of C3, C4, C4a, C5b-9/MAC, and factor H in the plasma and CSF are closely related to the severity of disease [166,167,168,169,170] upregulation of C1q, C3b, C4d, C3aR, C5aR, C5b-9 (MAC) in postmortem MS brains [175,176,177]	MAC exerts a direct impact on nervous tissue destruction [175,177] inhibition of MAC and C5aR1 reduced inflammatory gene expression and suppressed disease progression in EAE mouse model [179] C1q and C3 were deposited at synapses [181,182] the prominent role of C3 in synapse elimination [185] genetic deletion of C3 reduced the EAE clinical score and preserved synaptic density [186] genetic deletion of factor B delayed onset and reduced severity of EAE symptoms [187,188]
Epilepsy	increased expression of genes involved in the complement pathway [199,200] elevated level of components C1q, C3, C4, and C5b-9/MAC in resected brain samples [199,201,203,204]	elevated level of C1q, C3, C4, and C5b-9/MAC in animal models of epilepsy [199] long-lasting activation of complement C1q-C3 signaling in the hippocampus correlates with seizure frequency [205] C1q and C3b were involved in epileptogenic remodelling of the synaptic course and limit synaptic connectivity [211,212,213] inhibition of C1q showed some neuroprotective effects, but had no effect on memory deficit [210] inhibition of the C5aR1 receptor presented beneficial effect (diminished seizure power, protected hippocampal neurons from degeneration [209]. CFH may contribute to epileptogenesis (downregulation of CHF promoted acute seizures) [208]
Schizophrenia	abnormal activation in the classical complement pathway in sera of SZ patients. The function of several components has not been clarified yet. higher expression of C4A gene in brains (post-mortem) [216] C4a gene and elevated C4A expression predispose patients to SZ [216] the presence of C4 protein in synapses may be involved in dysfunction of synapse elimination. the altered level of C5 may be associated with decreased cortical thickness [241,242]	Not available
Autism	increased frequencies of *C4B* alleles in autistic patients and their mothers [253] elevated levels of C1q and C3 in the plasma of children with ASD [254,255]	deficiency of C3 in prefrontal cortex resulted in social deficits and repetitive behaviour in mice [217]

## Data Availability

Data sharing not applicable.
